# Global Challenges with Oral Antivirals for COVID-19

**DOI:** 10.1089/pop.2022.0171

**Published:** 2022-10-31

**Authors:** Dallas J. Smith, Avi J. Hakim, Andrea Taylor, Sarah D. Bennett, Pragna Patel, Ashley Greiner, Barbara J. Marston

**Affiliations:** 1 Epidemic Intelligence Service, Centers for Disease Control and Prevention, Atlanta, Georgia, USA.; 2 COVID-19 Response International Task Force, Centers for Disease Control and Prevention, Atlanta, Georgia, USA.; 3 Duke Global Health Innovation Center, Duke Global Health Institute, Duke University, Durham, North Carolina, USA.

**Keywords:** COVID-19, antivirals, global health, health care infrastructure

## Abstract

Oral antivirals for COVID-19 can be game changers in low- and middle-income countries (LMICs). Challenges that may hinder current and future oral antiviral rollouts span use in special populations, drug–drug and herb–drug interactions, adverse events, development of resistance, black markets, and equity in access and prescribing. Future antivirals may address some of these barriers; however, health systems around the world should be equipped to receive and administer COVID-19 oral antivirals. Improvements in manufacturing capacity, community engagement, capacity for testing and linkage to care, and systems for surveillance and safety monitoring could “change the game” for LMICs, irrespective of any specific antiviral drug. Investments in health care infrastructure can promote resilience, not only for COVID-19 but also for future local and global health crises.

## Are COVID-19 Oral Antivirals Game Changers in Low- and Middle-Income Countries?

Oral antivirals to treat COVID-19 could alter the trajectory of the pandemic by reducing severe disease, death, and health system strain, but there are significant barriers to their availability and effective use in low- and middle-income countries (LMICs), which are home to 85% of the world’s population. Prompt action by national and international leadership can address these barriers to unlock the potential impact of these drugs around the world.

### Oral antivirals are a critical tool

Since January 2020, the COVID-19 pandemic has evolved due to emergence of new SARS-CoV-2 variants, the development of natural and vaccine-derived immunity, and the availability of therapeutics. As of late April 2022, more than 500 million cases and more than 6 million deaths have been reported to the World Health Organization (WHO) while 59% of the global population has received the primary series of a COVID-19 vaccine.^1,2^ The development and rollout of effective COVID-19 vaccines have led to reduced mortality; however, inequities in access and issues with vaccine demand and hesitancy continue to stall vaccination program progress, and morbidity and mortality due to SARS-CoV-2 infection remain significant.^3^

Two oral antiviral treatments for COVID-19, nirmatrelvir/ritonavir and molnupiravir, have recently been approved in multiple countries and other antivirals are undergoing trials. When provided within 5 days of illness onset to persons with mild disease, nirmatrelvir/ritonavir reduced hospitalizations and death by 88%. Initial clinical trial data demonstrated molnupiravir to be 30% effective at preventing hospitalization and death, though preliminary data from India have shown real-world effectiveness of 65%.^4–6^

The emergence of new SARS-CoV-2 variants, including Omicron, that have mutations in the spike protein has impacted the efficacy of vaccines and therapeutics.^7^ Several monoclonal antibody preparations have been shown to be ineffective against the Omicron variant.^8^ Because nirmatrelvir/ritonavir and molnupiravir target the SARS-CoV-2–3CL protease and SARS-CoV-2-RNA–dependent RNA polymerase enzyme, respectively, these treatments are expected to maintain their effectiveness in the face of future variants.^9,10^

Moreover, these new oral antiviral drugs also offer logistical benefits over monoclonal antibodies, as tablets are easily transported and self-administered. Unlike monoclonal antibodies, the majority of which are administered through infusion in a health care setting, oral antivirals can be taken at home, reducing barriers to distribution and use. WHO has conditionally approved molnupiravir for high-risk individuals but recommends a host of harm mitigation measures such as pregnancy tests and pharmacovigilance programs. The health agency also issued a strong recommendation for nirmatrelvir/ritonavir in patients with nonsevere illness at the highest risk of hospitalization and conditionally recommends it in patients with nonsevere illness at a low risk of hospitalization.^11^

#### Special populations.

There are, however, important safety and implementation issues that require robust health infrastructure to effectively implement the use of these treatments. Owing to mutagenicity and teratogenicity concerns, molnupiravir is not recommended in women who are pregnant or breastfeeding, and birth control should be assured for women of reproductive age and, because the drug could be transferred in semen, for their male partners.^11^

The limited availability of birth control in many LMICs may preclude the use of molnupiravir for sexually active reproductive-age people. Oral antivirals may also have the potential to interact with oral contraceptives such as ethinyl estradiol, decreasing their effectiveness for pregnancy prevention.^12^ Backup nonhormonal contraception, such as condoms, during oral antiviral treatment may be warranted.

#### Drug–drug interactions.

The clinical trials for COVID-19 oral antivirals included a limited, relatively healthy population while evaluating their effectiveness.^4,5^ Patients with certain disease states, including HIV and tuberculosis, were excluded from the study; nirmatrelvir/ritonavir can have clinically harmful interactions with medications used to treat these conditions mainly from ritonavir’s potent CYP3A4 inhibition. In addition, patients taking medications to treat common noncommunicable diseases such as diabetes, hyperlipidemia, and cancer were not eligible to participate in the studies, but there are drug–drug interactions between these drugs and oral antivirals that require a change of dose, alternative therapy, or temporary discontinuation of a chronic medication.^11^

Some patients may also be taking herbal or other traditional treatments that could be involved in interactions. A thorough medication review is needed before dispensing, which may be difficult if patients do not know the names of medications they are taking, or medical charts are not readily available. Patients with chronic conditions are often at highest risk of severe COVID-19 and would warrant the use of oral antivirals after a positive COVID-19 test.

#### Adverse events.

Both nirmatrelvir/ritonavir and molnupiravir had promising side effect profiles in clinical trials; however, the side effect profiles might differ when used in an uncontrolled environment and provided to a wider range of patients.^4,5^ Caution, and in some cases dose adjustment, is required when administering these treatments to individuals with renal or hepatic insufficiency, but testing is not always available. The combination of nirmatrelvir/ritonavir and HIV medications containing ritonavir may cause an increase in gastrointestinal issues; however, patients with HIV were excluded in clinical studies. Given the short duration of treatment, such side effects may be well tolerated but data are lacking.

#### Drug resistance.

The risk that the use of oral antivirals will lead to selection of mutations in SARS-CoV-2 that confer resistance is unknown but warrants consideration given experiences with the treatment of other viral illnesses such as influenza and HIV. A particular concern is that monotherapy in immunocompromised persons could promote selection of a drug-resistant virus that could be transmitted to others. Substantial resistance has emerged to oral antivirals when they are taken as monotherapy for the influenza virus for a duration similar to that recommended for SARS-CoV-2 oral antivirals.^13,14^ There is limited information about using combination therapy to treat COVID-19, but *in vitro* combination therapy of oral antivirals has shown promise and may help mitigate potential resistance.^15^

Despite these concerns, oral antivirals will have an important role in the next phase of the pandemic, as more countries shift focus from emergency response to policies for “living with” COVID-19. Protecting the most vulnerable will require clinical and public health interventions in the face of ongoing transmission, low or waning natural and vaccination-induced immunity, and fewer or less stringent mitigation policies. Oral antiviral treatment may be a critical tool for transitioning to endemicity as hospitalizations are expected to decrease substantially.

### Challenges to global access

An ideal oral therapeutic would (1) be safe and effective, (2) maintain activity across variants, (3) have a substantial barrier to developing resistance, (4) be affordable, and (5) be accessible, including in LMICs. There will be challenges to accessing oral antivirals in LMICs, just as there have been for COVID-19 vaccines and other COVID-19 therapeutics.^3^ This is due to supply, funding, distribution, and uptake challenges.

#### Supply challenges.

So far, 99% of purchases made for COVID-19 oral antivirals have been by high-income countries.^16^ The Global Fund and UNICEF have both announced agreements with Pfizer for purchases of nirmatrelvir/ritonavir for use in LMICs (10 million and up to 4 million courses, respectively), but logistics and distribution plans are not yet finalized.

Promising developments through the Medicines Patent Pool (MPP) were announced for oral antivirals; these aim to reduce costs, increase supply, and improve access through sublicenses with generic manufacturers globally.^17,18^ The Access to COVID-19 Tools Accelerator financing framework, an initiative by WHO and other partners, anticipates costs for oral antivirals at 10 US dollars (USD) per course, which contrasts with higher prices paid in high-income countries (up to 700 USD per course).^19^ Other cost estimates put the price of molnupiravir between 5 and 50 USD.^20^ This lower price estimate may still potentially be prohibitive to LMICs for country-wide distribution.

However, timelines for the supply of generic medicines through the MPP licensure deals are long. Given the need for bioequivalence studies and WHO prequalification (WHO PQ), it could be a year before nirmatrelvir/ritonavir generics are produced in significant numbers. The generic manufacturing companies listed in the MPP deal will need to invest time and resources into obtaining WHO PQ. The interest and willingness to invest will depend on assured demand in LMICs to support an affordable and sustainable price from generic manufacturers participating in the agreements. Without large advance market purchases, generic manufacturers may opt out from producing these drugs.

In addition, nearly half of the world’s population live in countries excluded from the Pfizer and Merck MPP agreement.^17,18^ For upper-middle-income countries such as Argentina, Brazil, and Thailand, the oral antivirals may be priced out of reach without an option to purchase cheaper generics from the manufacturing companies listed in the MPP agreements. If the market follows trends seen in COVID-19 vaccine purchases, even those upper-middle-income countries that do make purchases may struggle to compete with high-income countries to obtain 2022 delivery from Pfizer and Merck.^16^

#### Distribution and uptake challenges.

Even if LMICs are able to secure supply and robust distribution systems are in place, costs of accessing the drugs could still limit whether patients can get them. Administration of oral antivirals is currently only indicated for persons who have a positive COVID-19 test (antigen or polymerase chain reaction) within 5 days of symptom onset. Therefore, robust test and treat strategies will be needed to facilitate timely testing, minimize time for return of results, and rapid initiation of treatment with an available oral antiviral.

National strategies will need to be tailored to the country and local context to implement solutions to address gaps in health care human resources and ensure there is demand for early testing and willingness to take treatment. When tests and medications are in limited supply, national strategies may need to focus on populations at highest risk for severe COVID-19 that may benefit the most from treatment.^11^ When supplies of oral antivirals are limited, providers could limit prescribing, saving antivirals for those at greatest risk or persons prioritized for other reasons.

Clinical considerations including side effects and drug–drug interactions and requirements for use in special populations could compound hesitation among providers to prescribe antivirals. In addition, underfunded pharmacovigilance programs in LMICs may not have the resources to adequately monitor for adverse events, which could further our understanding of the safety of these agents.

Hesitancy and mistrust are also potential barriers to the use of COVID-19 treatments. The hesitancy that has hindered COVID-19 vaccine rollout could carry over to oral antivirals. Already, up to 30% of persons eligible for oral antivirals are refusing them in high-income countries such as Israel.^21^

Unregulated and counterfeit versions of the current oral antivirals have been identified in black markets globally.^22,23^ The driving forces behind this black-market demand likely include limited supplies and the practice of self-medication where access to primary care providers is difficult. Unregulated and fake versions of oral antivirals can delay proper treatment, lead to severe health outcomes from COVID-19 and from the counterfeit drug, and may increase distrust and hesitancy in oral antivirals over time.

### How can the impact of COVID-19 treatments be optimized?

Investment in more resilient health systems now, while LMICs are waiting for oral antivirals, can start to address the aforementioned issues before they become barriers to access. Strategic and equitable test-to-treat guidelines, educational and awareness campaigns, and pharmacovigilance systems must be established and improved so that global supply of COVID-19 oral antivirals can be effectively used (Table 1).

Test-to-treat strategies can draw on existing human resources for health and innovative models to expand access to oral antivirals. Task shifting can help facilitate administration of these treatments while strengthening health care systems long term.^24^ Development of standardized treatment protocols will facilitate task shifting, allowing nurses, pharmacists, and community health workers to perform COVID-19 testing and dispense oral antivirals. Telehealth enabled by self-testing and use of mobile phones may prove effective in some settings. Results of self-tests could be shared with providers or pharmacists by video or photograph.

Innovations in delivering oral antivirals, such as fast-track pharmacies, designated community distribution sites, and delivering oral antivirals to prioritized at-risk individuals or facilities (eg, nursing and long-term care homes) before diagnosis can accompany task-shifting and telehealth initiatives.^24^

Ensuring test-to-treat services for oral antivirals to populations most affected by inequalities (rural, minority, and other underserved populations) through community health workers and telehealth may be helpful in overcoming inequities.^25^ Community and faith-based institutions can be mobilized to identify pockets of populations suffering most from gaps in health care access and provide grass-root support for oral antiviral uptake.

Educational campaigns and job aids with clear prioritization guidelines from trusted sources can equip providers with the tools to promote equitable practices and assist with prescribing oral antivirals. Ministries of health, regulatory authorities, and academic institutions could consider developing a comprehensive resource of commonly used conventional and herbal medications in their country and outline potential interactions with COVID-19 therapeutics. Providers can use these resources, in written or electronic form (ie, smart phone applications), to clinically evaluate interactions, monitor for adverse effects, or change therapeutic regimens.

Other educational outreach about the harms of counterfeit medications, sustained investment in local manufacturing, deterring illegal activity of counterfeit medications through legislative reform, and, most importantly, equitable access to regulated medications can help thwart a bourgeoning black market globally.^26^

Governments and nongovernmental institutions can work with pharmaceutical industries and partners to invest in and strengthen pharmacovigilance programs to monitor for adverse events and drug–drug interactions to improve our understanding of the safety profiles for oral antivirals and future therapeutics beyond the more limited clinical trial data. For newly established pharmacovigilance programs, a platform initially built around populations at high COVID-19 risk could provide great benefit, as these populations may be at greatest risk of adverse events and drug–drug interactions. Frequent prescription and medical record audits can actively identify adverse events, and training of medical professionals on pharmacovigilance programs will assist in identifying harm from oral antivirals.^27^

The global influenza neuraminidase inhibitor susceptibility network could be used to actively monitor sequences for resistance, particularly in individuals with immunosuppressing conditions.^28^ Future clinical trials could build on the promising activity combination therapy has shown in *in vitro* studies; combination therapy could mitigate the risk of drug resistance.^15^

### Building resilient health systems

Future generations of oral antivirals may address some of the challenges described herein with the first generation anti-COVID-19 antivirals. For example, S-217622 produced by Shionogi may not need to be combined with a pharmacokinetic booster and is likely subject to fewer drug–drug interactions.^29^ LAU-7b by Laurent Pharmaceuticals may have a role in both mild and severe disease and is not restricted to use within a limited time frame.^30^

Although new drugs are being developed, governments, civil society, multilateral institutions, and donors should work in a coordinated way, decoupled from politics, to ensure that health systems around the world are equipped to receive and administer COVID-19 oral antivirals. Without these interventions and resources, COVID-19 therapeutics could be underutilized, ineffective, or harmful.

Surveillance systems for adverse events and resistance, educational campaigns for providers, and plans to increase provider and public demand while overcoming hesitancy can lead to successful treatment efforts. In turn, these efforts could make manufacturers more confident that they will receive future orders of oral antivirals. If these interventions are missing, global supply could suffer if generic manufacturers opt out of producing oral antivirals and high-income countries purchase the available supply at costs unaffordable to LMICs.

The global community has acknowledged that distribution of vaccines does not necessarily translate to people being vaccinated. Factors such as median per capita income, human development index, percentage of those who used the internet in the prior 3 months, and health expenditure have been associated with global success of COVID-19 vaccination rollouts; countries with lower levels of these indicators may warrant particular attention for rollout of therapeutics.^3^

Strengthening LMICs’ capacity to overcome hurdles such as lack of infrastructure and mistrust during every step of the rollout process is vital for biomedical interventions.^31^ Variants of SARS-CoV-2 or other pathogens will likely emerge in future; the investments made in the rollout of COVID-19 vaccines and antivirals can provide a sustainable platform for use, including during future public health emergencies.

Improvements in manufacturing capacity, community engagement, capacity for testing and linkage to care, and systems for surveillance and safety monitoring are all needed. Progress in these realms could “change the game” for LMICs, irrespective of any specific antiviral drug. Investments in infrastructure can promote health system resilience, not only for COVID-19 but also for future local and global health crises.

## Figures and Tables

**Table 1.** COVID-19 Oral Antiviral Challenges and Opportunities

Challenges	Opportunities
Equitable antiviral access and prescribing	Strategic and equitable test-to-treat guidelines
	Task shifting to nurses, pharmacists, and community health workers
	Standardized treatment protocols
	Telehealth visits
	Innovative oral antiviral delivery such as fast-track pharmacies, designated community distribution sites, and delivering oral antivirals to prioritized at-risk individuals or facilities (eg, nursing and long-term care homes) before diagnosis
	Mobilization of community and faith-based institutions
	Educational campaigns and job aids with clear prioritization guidelines from trusted sources
Drug-drug and herb-drug interactions	Comprehensive resource of commonly used conventional and herbal medications that interact with oral antivirals
Adverse events	Targeted investment in and strengthening of pharmacovigilance programs
	Frequent prescription and medical record audits
Black market	Educational outreach about the harms of counterfeit medications
	Sustained investment in local manufacturing
	Deterring illegal activity of counterfeit medications through legislative reform
	Equitable access to regulated medications
Drug resistance	Active monitoring of sequences through global influenza neuraminidase inhibitor susceptibility network or similar platform
	Further research on combination antiviral therapy
